# Comparison between Platelet Lysate, Platelet Lysate Serum, and Fetal Bovine Serum as Supplements for Cell Culture, Expansion, and Cryopreservation

**DOI:** 10.3390/biomedicines12010140

**Published:** 2024-01-09

**Authors:** Juan Manuel Duarte Rojas, Luz Marina Restrepo Múnera, Sergio Estrada Mira

**Affiliations:** 1 Tissue Engineering and Cellular Therapies Group—GITTC, Faculty of Medicine, University of Antioquia, Medellín 050010, Colombia; marina.restrepo@udea.edu.co (L.M.R.M.); sergio.estrada@udea.edu.co (S.E.M.); 2 Biomedical Basic Sciences Academic Corporation, University of Antioquia, Medellín 050010, Colombia; 3 Cellular Therapy and Biobank Laboratory, Hospital Alma Mater de Antioquia, University of Antioquia, Medellín 050010, Colombia

**Keywords:** fetal bovine serum (FBS), human platelet lysate and platelet lysate serum, mesenchymal stem cells (MSCs), fibroblasts, culture, cryopreservation

## Abstract

As cell culture supplements, human platelet lysate (PL) and human platelet lysate serum (PLS) are alternatives to fetal bovine serum (FBS) due to FBS-related issues such as ethical concerns, variability between batches, and the possible introduction of xenogenic contaminants. This study compared the composition and efficacy of PL, PLS, and FBS as supplements in the culture and cryopreservation of human dermal fibroblasts, Wharton’s jelly-derived mesenchymal stem cells (WJ-MCS), and adipose tissue (AdMSC). Biochemical components, some growth factors, and cytokines present in each of them were analyzed; in addition, the cells were cultured in media supplemented with 5% PL, 5% PLS, and 10% FBS and exposed to different freezing and thawing solutions with the supplements under study. Biochemical parameters were found to be similar in PL and PLS compared to FBS, with some differences in fibrinogen and calcium concentration. Growth factors and cytokines were higher in PL and PLS compared to FBS. Cell proliferation and morphology showed no significant differences between the three culture media. Regarding the cryopreservation and thawing of cells, better results were obtained with PLS and FBS. In conclusion, PL and PLS are an excellent choice to replace the standard supplement of animal origin (FBS) in the media used for the culture and cryopreservation of fibroblasts, WJ-MSC, and AdMSC.

## 1. Introduction

In the last two decades, there has been a worldwide boom in regenerative medicine. As a result, advanced treatments have emerged, such as cell therapies based on mesenchymal stem cells or fibroblasts, which are used successfully in multiple diseases [1]. Expanding and cultivating these cells requires specific culture media that contain nutrients and essential bioactive molecules that facilitate the growth of the cells without altering their normal physiological properties [2,3,4].

Mesenchymal stem cells (MSCs) have great therapeutic potential due to their ability to differentiate into different cell types, modulate the immune response, and reduce inflammation [1,5]. The role that these cells can play in treating a wide variety of diseases (cardiac, metabolic, neurodegenerative, autoimmune disorders, among others) has been investigated. These cells have been isolated from various body tissues such as bone marrow, adipose tissue, and the umbilical cord [2,5,6,7,8].

Fibroblasts are responsible for the production of collagen, elastin, and other structural proteins found in the extracellular matrix. These proteins are essential because they give tissues integrity and strength and allow them to maintain their elasticity and flexibility. Fibroblasts play an important role in wound healing and repair of damaged tissues, and for this reason, have been widely used in medical research and cell therapy [9,10].

Large-scale cell production is a critical step in the development of safe and effective advanced therapies. For this, culture methods are sought that guarantee high yield, viability, reproducibility, and safety of the cell product [2,11]. Cells are obtained using supplemented culture media that favor their expansion. One of the most widely used supplements for cell culture is fetal bovine serum (FBS), which serves as a source of hormones, growth factors, amino acids, proteins, vitamins, inorganic salts, trace elements, carbohydrates, lipids, and other factors necessary for the metabolism and normal proliferation of cells [12,13,14].

Since its introduction in the 1950s, FBS has been used as a universal source of growth factors and essential nutrients for cell adhesion, proliferation, differentiation, and maintenance in most cell cultures [11,13,14,15,16,17]. Nonetheless, the growing use of this additive has led to concerns about the welfare of the animals that it comes from; its costs and complex supply chain; and its undesirable effects, such as the potential for immunogenicity, the presence of xenogeneic antigens, and the spread of prions and other diseases of animal origin. These concerns have been raised in both the scientific and ethical debates on the preparation, distribution, and use of FBS [3,4,11,13,14]. This has led to the search for alternative supplements for the growth media used in cell culture and therapies [1,2,4,11,14,15]. Some of the supplements that can serve as an alternative to FBS as a source of specific nutrients for cells are derived from human blood components, serum-free media, and proteins of animal origin [1,4,11,14,18].

Blood components such as plasma, serum, and platelet derivatives are used to supply the nutritional needs of cells in culture, both in research and in cell therapies [1,2,4,11,17]. Platelet derivatives have been extensively studied and are used to supplement different types of culture media and nourish various types of cells, due to the role they play in a range of immunomodulatory, proliferative, cell adhesion, and protein synthesis functions necessary for the proper function of cells in culture [3,19,20,21].

Furthermore, platelet derivatives demonstrate clear advantages compared to FBS as products for the ex vivo culture and expansion of different cell types. This is due to the possibility of highly standardized large-scale manufacturing, facilitated by the use and donation of expired allogeneic platelet units unsuitable for transfusion, as well as being a non-animal source of a variety of biochemical components and growth factors, thus reducing the risks encountered and described above with FBS. Likewise, cell proliferation during culture with platelet derivatives facilitates the sufficient production of safe cellular therapies within an appropriate timeframe [22].

Platelets are nucleated cell fragments that are derived from the cytoplasm of megakaryocytes in the bone marrow and play an essential role in primary hemostasis, wound healing, inflammation, immunity, and tissue regeneration. Due to their short half-life (8 to 10 days), around 15–40 × 10^9^ platelets must be produced daily to maintain a normal blood count of 1.5–4.5 × 10^5^ /mL [17,23].

Platelets contain various organelles such as mitochondria, peroxisomes, and ribosomes, as well as glycogen, and alpha, dense, and lambda granules. The rupture of these releases coagulation factors, chemokines, proteins, immune molecules, and other bioactive components in the form of human platelet lysate (PL) [17,21,24,25,26]. This product contains a variety of nutrients and proteins such as albumin, folate, vitamin B12, glucose, triglycerides, cholesterol, immunoglobulins, and other factors that contribute to balancing the colloidal pressure of the culture medium [1,17].

Some of the factors that make up platelet lysate are platelet-derived growth factor (PDGF—AA, AB, and BB isoforms), transforming growth factor beta (TGF-β), insulin-like growth factor-1 (IGF-1), brain-derived neurotrophic factor (BDNF), vascular endothelial growth factor (VEGF), epidermal growth factor (EGF), basic fibroblast growth factor (FGF-b or FGF-2), hepatocyte growth factor (HGF), connective tissue growth factor (CTGF) and bone morphogenetic protein-2, -4 and -6 (BMP-2, -4, -6) [16,17,24,25]. Therefore, PL is proposed as a suitable supplement for culture media due to its ability to provide essential nutrients and growth factors. Its use can improve the quality and safety of cell cultures both for biomedical research and for cell therapies [1,2,3,17].

This work aimed to characterize the compositions of human platelet lysate and human platelet lysate serum and compare these with fetal bovine serum. Additionally, it sought to evaluate their use as a supplement for the culture and cryopreservation of human dermal fibroblasts and mesenchymal stem cells derived from Wharton’s jelly and adipose tissue.

## 2. Materials and Methods

### 2.1. Obtaining Platelet Units

Platelets not suitable for transfusion due to their low leukocyte count were donated by the blood bank of the School of Microbiology of the University of Antioquia. The approximate volume per unit was 50 to 65 mL. These were preserved with citrate phosphate dextrose adenine 1 (CPDA-1) anticoagulant. It was verified before use that the units were negative for HTVL-1, hepatitis B virus, antibodies against core hepatitis B, HIV 1–2, hepatitis C virus, syphilis, and Chagas disease. The units were stored at −20 °C from the time they were obtained from the blood bank until their use.

### 2.2. Preparation of Platelet Pools

Three hundred units of platelets of blood group O and Rh factor + and − frozen at −20 °C were taken, divided into three batches of 100 units of platelets and thawed at a temperature between 2 and 8 °C for 24 h. They were transferred to a thermoregulated bath at 37 °C for 1 h. To prepare each batch of platelet pools, 5 mL of each unit was taken and combined in sterile 500 mL vials. Once each platelet pool was obtained, it was stored at −20 °C until use.

### 2.3. Obtaining Platelet Lysate and Platelet Lysate Serum

**Figure 1:**
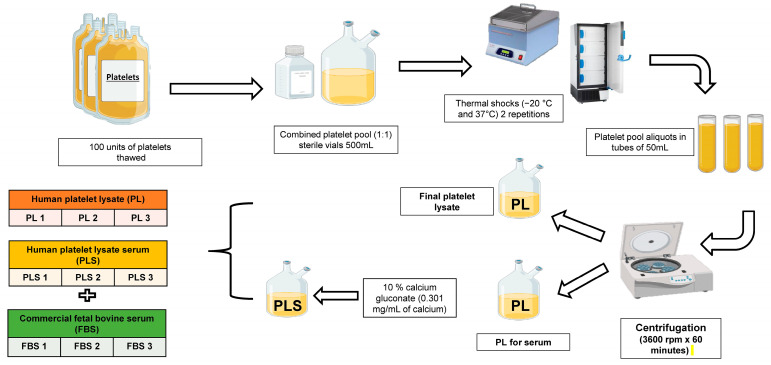
Preparation of platelet lysate and human platelet lysate serum from platelet units.

To obtain PL, a 500 mL unit of each platelet pool was thawed and then subjected to heat shock with two cycles of thawing (37 °C) and freezing (−20 °C) to induce platelet lysis. After this, 250 mL of each batch of lysate was taken and aliquoted into 50 mL tubes, which were then centrifuged at 3600 rpm for 1 h at room temperature. The supernatant or platelet lysate was taken for subsequent filtering and storage in 35 mL aliquots at −20 °C until use (Figure 1).

To obtain human platelet lysate serum (PLS), the remaining 250 mL was taken, and coagulation was induced with 8 mL of 10% calcium gluconate (B. Braun, Melsungen, Germany). Once coagulation was achieved, the platelet lysate serum was recovered and stored in the same way as the PL, as described above.

### 2.4. FBS Storage

Units of 3 different commercial batches of FBS (inactivated Gibco, Waltham, MA, USA; Biowest, Lakewood Ranch, FL, USA) were purchased, thawed at 2–8 °C for 24 h, and stored in 45 mL aliquots at −20 °C until use (Figure 1).

### 2.5. Biochemical Analysis of PL, PLS, and FBS

The concentrations of the following biochemical components were measured in each of the PL, PLS, and FBS batches: total protein, albumin, total cholesterol, low-density lipoprotein (LDL), high-density lipoprotein (HDL), triglycerides, glucose, transferrin, fibrinogen, ferritin, calcium (Ca^2+^), magnesium (Mg^2+^), iron (Fe^3+^), chloride (Cl^−^), sodium (Na^+^), potassium (K^+^), total cortisol, insulin, total triiodothyronine (T3), total thyroxine (T4), thyroid-stimulating hormone (TSH), and immunoglobulin G (IgG). These analyses were carried out in the clinical laboratory of the School of Microbiology of the University of Antioquia.

### 2.6. Growth Factor and Cytokine Analysis

The concentrations of the following growth factors were evaluated by ELISA (ELK Biotechnology—Biolake, Donghu New & High Technology Development Zone, Wuhan, China): PDGF-AB (pg/mL, IGF-1 (ng/mL); EGF (pg/mL), FGF2 (pg/mL), TGF-ß1 (pg/mL). The assay was performed in triplicate on each of the 3 batches of PL, PLS, and FBS.

Additionally, the concentrations of the following cytokines and growth factors were evaluated by Luminex (MILLIPLEX Human Cytokine/Chemokine Magnetic Bead Panel—Immunology Multiplex Assay, Luminex xMAP technology, Merck, Darmstadt, Germany): interleukin 6 (IL-6), interleukin 10 (IL-10), the chemokine CCL5 or RANTES, platelet-derived growth factor isoform AA (PDGF-AA), vascular endothelial growth factor A (VEGF-A), tumor necrosis factor-alpha (TNF-α), receptor antagonist interleukin 1 (IL1RA), granulocyte-macrophage colony-stimulating factor (GM-CSF), and granulocyte-colony-stimulating factor (G-CSF). The evaluation was performed in duplicate on each of the 3 batches of PL, PLS, and FBS.

### 2.7. Isolation and Culture of Dermal Fibroblasts

Retro auricular skin biopsies were taken with a 5 mm punch from two healthy volunteers after they had signed the informed consent. To obtain dermal fibroblasts, the explant technique was used, in which 1 mm^2^ skin fragments were deposited in 25 cm^2^ dishes, then cultured with DMEM/Ham-F12 (Lonza, Walkersville, MD, USA) mixed medium supplemented with 10% FBS (Biowest), 100 U/mL penicillin (Lonza), 100 mg/mL streptomycin (Lonza) and 1% L-glutamine (Lonza) until the necessary number of cells for subsequent assays was obtained.

### 2.8. Isolation and Culture of Wharton’s Jelly Mesenchymal Stem Cells (WJ-MSCs)

Two umbilical cords were obtained from healthy pregnant mothers after they had signed the informed consent. These were cleaned, and fragments of Wharton’s jelly were taken, and the explants were individually sown in 25 cm^2^ dishes with DMEM/Ham-F12 culture medium with 10% FBS, 100 U/mL penicillin, 100 mg/mL streptomycin, and 1% L-glutamine until the required number of cells for subsequent assays was obtained.

Mesenchymal cells were characterized by flow cytometry according to the standards of the International Society for Cellular Therapies [27].

### 2.9. Isolation and Culture of Adipose Tissue Mesenchymal Stem Cells (AdMSC)

Periumbilical liposuction was performed on two healthy donors after they had signed the informed consent. Between 20 and 30 mL of adipose tissue was taken and deposited in 50 mL tubes. Normal saline solution was added in a 2:1 ratio, and the sample was centrifuged at 1000 rpm for 10 min to remove red blood cells. Next, the adipose tissue was placed in a conical tube with type 1 collagenase (Gibco) at a concentration of 1 mg/mL in a 1:1 ratio and was orbital shaken at 37 °C for 2 h. The sample was passed through a 70 µm Cellstrainer (Falcon, Thermo Scientific, Waltham, MD, USA) and centrifuged at 1500 rpm for 5 min, after which the supernatant was discarded. The cell pellet was then resuspended in DMEM/F12 with 10% FBS, 100 U/mL penicillin, 100 mg/mL streptomycin, and 1% L-glutamine. The cells were cultured in 25 cm^2^ dishes until the required number of cells for subsequent assays was obtained. The mesenchymal cells were characterized by flow cytometry according to the standards of the International Society for Cellular Therapies [27].

### 2.10. Evaluation of PL, PLS, and FBS as a Supplement to the Culture Medium

A total of 2.3 × 10^4^ cells of each cell type, fibroblasts, AdMSC, and WJ-MSC were seeded per 9 cm^2^ well. They were cultured in DMEM/Ham-F12 supplemented with 5% PL, 5% PLS, or 10% FBS, together with 100 U/mL penicillin, 100 mg/mL streptomycin, and 1% L-glutamine. In the medium supplemented with PL, 1% sodium heparin (Fresenius Kabi, Toronto, ON, Canada) at 5000 IU/mL was added. Photographic records were taken at 4, 24, 48, 72, and 96 h, and 3 photos were taken for each well. Images were analyzed with Fiji, ImageJ software (2.9.0 version, Fiji). The assay was performed in triplicate.

### 2.11. Evaluation of PL, PLS, and FBS as Media for Cryopreservation

Totals of 6.7 × 10^5^ fibroblasts, 1.0 × 10^6^ WJ-MSC and 1.0 × 10^6^ AdMSC cells were taken and frozen separately with 90% PL, PLS, or FBS plus 10% dimethyl sulfoxide—DMSO (CryoSure, 99.99% DMSO; WAK-Chemie Medical GmbH, Steinbach, Germany). These were left in cryopreservation in liquid nitrogen for 1 week and then thawed in a thermoregulated bath at 37 °C. For thawing, medium supplemented with 30% PL, PLS, or FBS + 70% DMEM/Ham-F12 was used. Viability was evaluated before and after freezing with trypan blue (Table 1). For the freezing and thawing solution supplemented with PL, 1% sodium heparin at 5000 IU/mL was added.

Once the cells had thawed, 1.75 × 10^4^ cells of each cell type were seeded in 3.5 cm^2^ wells at a density of 5 × 10^3^ cells per cm^2^, using DMEM/Ham-F12 medium supplemented with 5% PL, penicillin at 100 U/mL, streptomycin at 100 mg/mL, 1% L-glutamine, and 1% heparin sodium. Cell adhesion and proliferation (% cell confluence) were assessed at 24, 48, and 72 h, and three photographs were taken for each well and analyzed using ImageJ software. The assay was performed in duplicate (Table 1). 

### 2.12. Statistical Analysis

For statistical analysis, GraphPad Prism software version 9.5 (La Jolla, CA, USA) was used. One-way or two-way analysis of variance (ANOVA) was applied for all values obtained from the trials, followed by a comparison between groups using Tukey’s test. Differences resulting in p values below 0.05 were considered significant (*p* < 0.05). In addition, the corresponding means and standard deviations were provided.

## 3. Results

### 3.1. Biochemical Composition of PL, PLS, and FBS

To evaluate the homogeneity of the composition of the three batches of PL and PLS prepared and compare these with the three commercial batches of FBS, the concentrations of 22 biochemical components, six growth factors, and eight molecules related to cytokines, interleukins, and chemokines were analyzed.

Table 2 shows the mean standard deviation (±SD) of the biochemical concentration of each of the components evaluated in the three different batches of PL, PLS, and FBS, in addition to the reference plasma values for each component. Fibrinogen concentration was significantly lower in PLS and FBS (<50 mg/dL test detection limit) than in PL (141 ± 56.5 mg/dL) (*p* < 0.0001), while transferrin, ferritin, and immunoglobulin G were found to be significantly higher in PL (206.28 ± 3.6 mg/dL; 89.62 ± 5.5 ng/mL; 1038 ± 11 mg/dL, respectively) and PLS (206.28 ± 9.4 mg/dL; 86.97±8.1 ng/mL; 1016.8 ± 31.4 mg/dL) than in FBS (0.1 ± 0.2 mg/dL; 1.04 ± 0 0.5 ng/mL, 4.59 ± 9.1 mg/dL) (*p* <0.0001). No differences were found in albumin and total protein concentrations.

The concentrations of total cholesterol, LDL, triglycerides, and glucose were significantly higher in the PL preparations (152.14 ± 5.7; 103.48 ± 2.5; 121.58 ± 16.1; 192.75 ± 23, 1 mg/dL, respectively) and PLS (141.81 ± 4.2; 96.25 ± 2.5; 103.51 ± 9.1; 189.75 ± 21 mg/dL) than in FBS (32. 58 ± 3.3, 20.4 ± 3.8, 49.58 ± 34.8, 69.25 ± 51.2 mg/dL) (*p* < 0.0001) (*p* < 0.005). The concentrations of Cl^−^ and Fe^2+^ were higher in the FBS samples (103.50 ± 50 mmol/L and 187.75 ± 20.7 μg/dL) than in those of PL (83.07 ± 2.4 mmol/L and 79.5 ± 2.6 μg/dL) and PLS (80.55 ± 2.5 mmol/L and 77.5 ± 5.3 μg/dL) (*p* < 0.0001). Na^+^ was higher in the PL and PLS preparations (>150 mmol/L). On the other hand, there was a statistically significant difference in the concentration of Ca^2+^ between the three types of supplements, with values for PLS of 36.04 ± 2.2 mg/dL, PL of 7.55 mg/dL and FBS of 14.44 ± 0.3 mg/dL. This difference can be attributed to the use of calcium gluconate to induce clot formation. There were no significant differences in the concentrations of K^+^ and Mg^2+^ (Table 2).

Finally, in the analysis of hormone concentration, total cortisol, insulin, and TSH showed significantly higher values in PL (7.28 ± 0.7 μg/dL; 2.61 ± 0.3 mIU/L and 1.72 ± 0.2 uIU/mL, respectively) and in PLS (7.73 ± 1.6 μg/dL; 2.57 ± 1.3 mIU/L and 1.71 ± 0.3 uIU/mL) than in FBS (0.05 ± 0.1 μg/dL; 0.06 ± 0.1 mUI/L and 0.03 ± 0.0 uIU/mL) (*p* < 0.0001). Meanwhile, thyroxine was significantly lower in PL and PLS (7.28 ± 0.3; 7.18 ± 0.3 mg/dL; *p* < 0.0001) than in FBS (15.6 ± 4.6 mg/dL). There were no differences in the concentration of triiodothyronine in the three samples (Table 2).

### 3.2. Growth Factor and Cytokine Analysis

**Figure 2:**
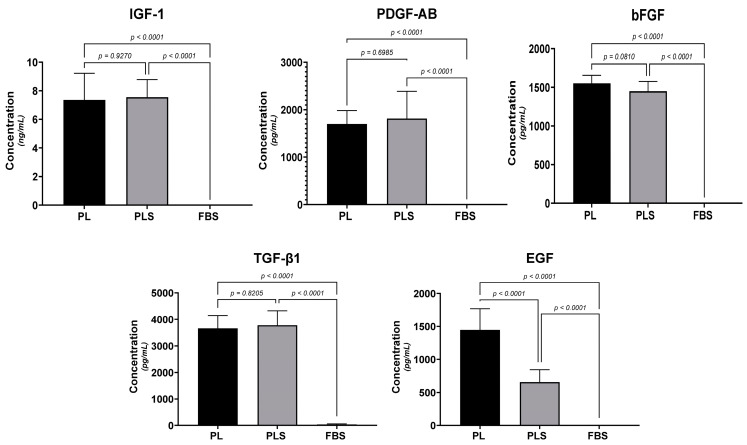
Comparison of the concentrations of the growth factors IGF-1, PDGF-AB, bFGF, TGF-β1, and EGF by the sandwich ELISA technique. Results are shown as the mean ± SD of the three batches of PL, PLS, and FBS. One-way ANOVA followed by Tukey’s test was used to statistically determine the differences.

When analyzing the growth factors by ELISA technique, it was observed that FGF-b, TGF-B1, PDGF-AB, and IGF-1 were significantly higher in all PL and PLS preparations than in FBS (*p* < 0.0001). The coagulation induced when obtaining the PLS did not influence the concentration of any of these growth factors in a statistically significant way; however, a lower concentration of EGF was observed in the PLS preparations than in PL (*p* < 0.0001) (Figure 2).

**Figure 3:**
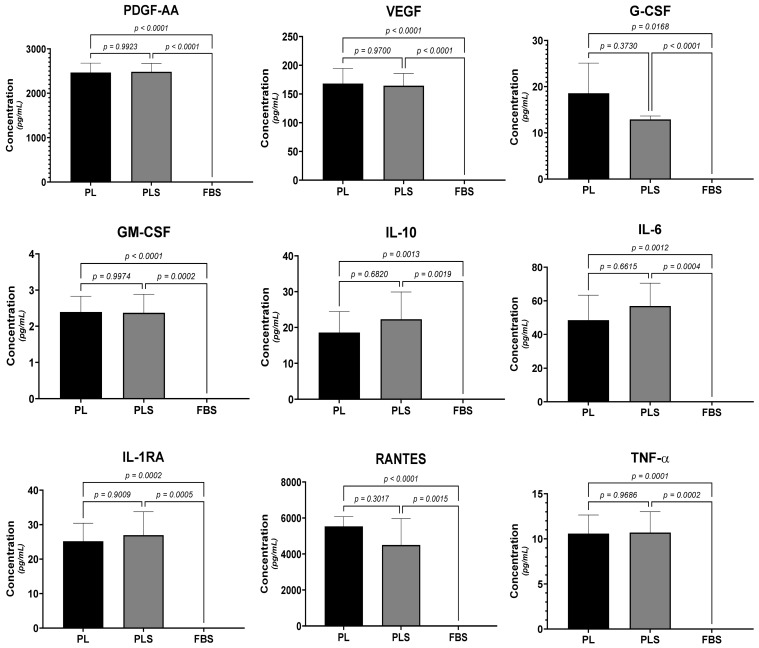
Comparison of the concentrations of growth factors and cytokines PDGF-AA, VEGF-A, G-CSF, GM-CSF, IL-10, IL-6, IL-1RA, RANTES, and TNF-α by Luminex technique. Results are shown as the mean ± SD of the three batches of PL, PLS, and FBS. One-way ANOVA followed by Tukey’s test was used to statistically determine the differences.

Regarding the concentrations of cytokines and growth factors (IL-6, IL-10, RANTES, PDGF-AA, VEGF-A, TNF-α, IL1RA, GM-CSF, and G-CSF) involved in cell stimulation and differentiation and the pro-inflammatory and anti-inflammatory responses, no significant differences were observed when comparing the PL vs. PLS (*p* > 0.05). On the other hand, when comparing PL and PLS with FBS, statistically significant differences were observed in the concentrations of all of the molecules analyzed (*p* < 0.005 PL vs. FBS; *p* < 0.005 PLS vs. FBS) (Figure 3).

### 3.3. Proliferation Kinetics of Fibroblasts, WJ MSCs and AdMSCs

**Figure 4:**
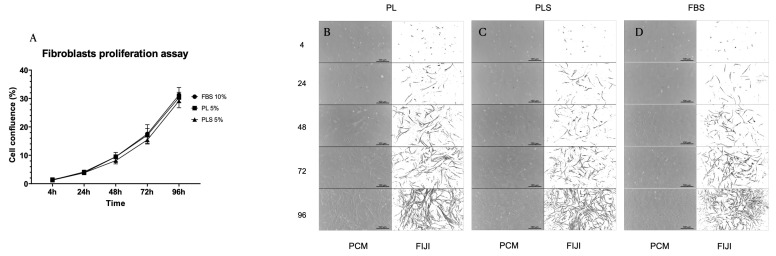
Proliferation kinetics behavior of fibroblasts with different culture media supplemented with PL, PLS, and FBS. (**A**) No differences were observed in the proliferation behavior of fibroblasts after 4, 24, 48, 72, and 96 h with the different culture media evaluated. Data are shown as mean values and standard deviation. Cell morphology was not affected by the medium supplemented with PL (**B**), PLS (**C**), and FBS (**D**).

**Figure 5:**
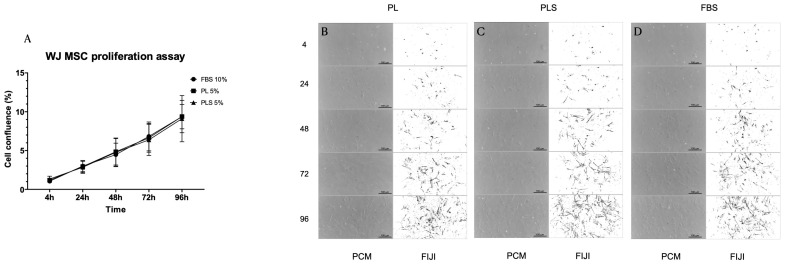
Proliferation kinetics behavior of WJ-MSCs with different culture media supplemented with PL, PLS, and FBS. (**A**) No differences were observed in the proliferation behavior of WJ-MSCs after 4, 24, 48, 72, and 96 h with the different culture media evaluated. Data are shown as mean values and standard deviation. Cell morphology was not affected by the medium supplemented with PL (**B**), PLS (**C**), and FBS (**D**).

**Figure 6:**
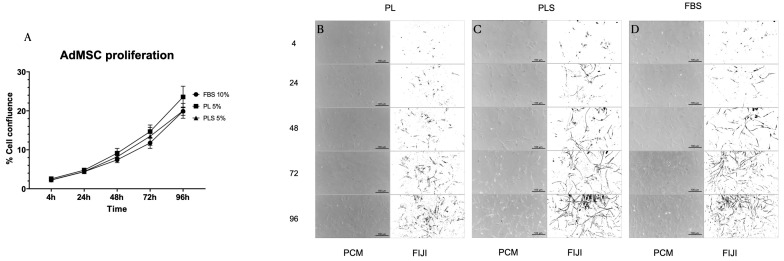
Proliferation kinetics behavior of AdMSCs with different culture media supplemented with PL, PLS, and FBS. (**A**) No differences were observed in the proliferation behavior of AdMSCs after 4, 24, 48, 72, and 96 h with the different culture media evaluated. Data are shown as mean values and standard deviation. Cell morphology was not affected by the medium supplemented with PL (**B**), PLS (**C**), and FBS (**D**).

When evaluating the proliferation kinetics of fibroblasts, WJ MSCs, and AdMSCs at 4, 24, 48, 72 and 96 h with the different culture media (DMEM/Ham-F12 + 10% FBS; DMEM/Ham-F12 + 5% PL; DMEM/Ham-F12 + 5% PLS), it was observed that after 96 h of culture, there were no significant differences in the cell proliferation curves with the three types of culture media evaluated. That is, the platelet derivatives presented the same behavior as the standard supplement of animal origin (FBS) in terms of culturing, maintenance, and growth of fibroblasts, WJ MSCs, and AdMSCs. Moreover, it should be noted that the concentrations used for PL and PLS were half that of FBS (Figure 4A, Figure 5A and Figure 6A).

Similarly, no changes were observed in the morphology of the cultured cells with the different treatments, as shown in Figure 4B–D, Figure 5B–D and Figure 6B–D.

### 3.4. Efficiency of PL, PLS, and FBS in Cell Cryopreservation

**Figure 7:**
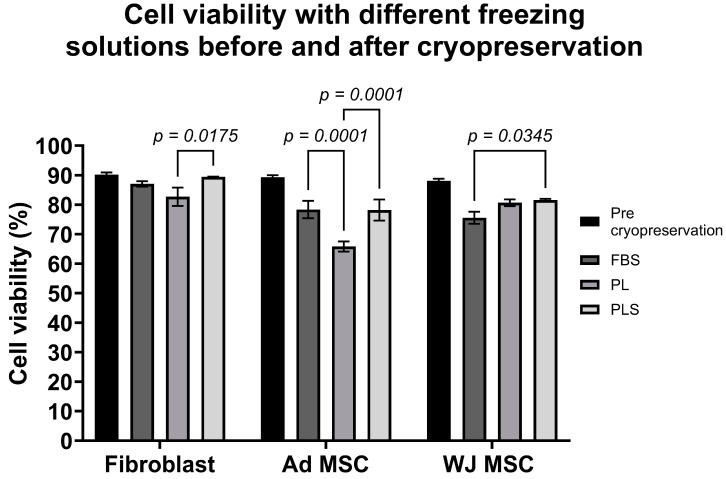
Comparison of pre- and post-freezing cell viability of fibroblasts, WJ-MSCs, and AdMSCs with PL, PLS, and FBS. Results are shown as the mean ± SD of the freezing solutions with PL, PLS, or FBS, and cell viability pre-cryopreservation. Two-way ANOVA followed by Tukey’s test was used to statistically determine the differences.

When comparing the freezing and thawing of fibroblasts, WJ-MSCs, and AdMSCs in solutions with PL, PLS, or FBS, it was observed that fibroblasts maintained post-thawing viability above 85% with all media. However, with PLS, the viability obtained was much higher than that with PL (Figure 7). In the case of WJ-MSCs, post-thawing viability was above 80% with all three supplements but was higher for the freezing solutions with PL (80.65%) and PLS (81.54%) than with FBS (75.56%, *p* < 0.05 comparing FBS vs. PLS) (Figure 7). AdMSCs maintained post-thawing viability above 75%, but this was lower than their pre-freezing viability (89.30%). In this case, the lowest yield was with PL, with a significant loss of viability, by close to 20% (65.80%; *p* < 0.005 PL vs. PLS), possibly due to the formation of fibrin networks during the freezing and thawing process that altered cell recovery after thawing (Figure 7).

**Figure 8:**
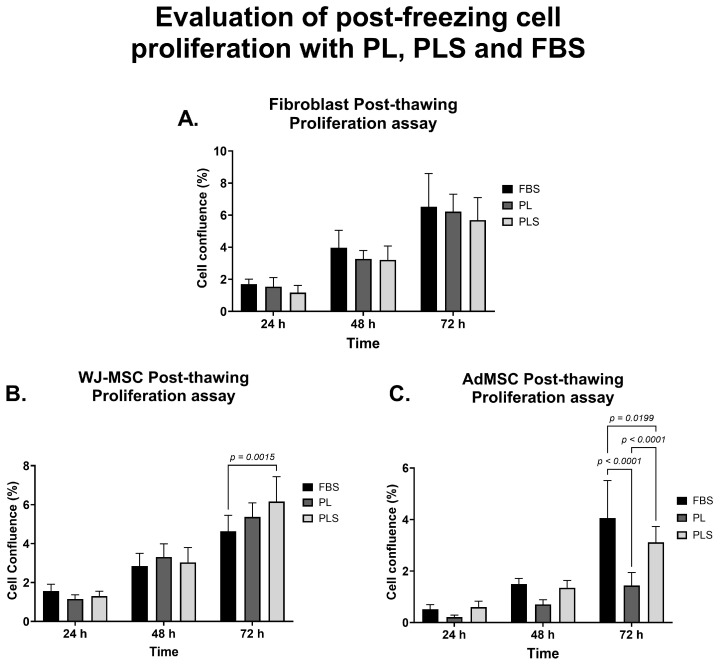
Evaluation of post-freezing cell proliferation of fibroblasts, WJ-MSCs, and AdMSCs with PL, PLS, and FBS. Proliferation kinetics of fibroblasts (**A**), WJ-MSCs (**B**), and AdMSCs (**C**) according to post-thawing between 24 and 72 h of culture. Data are shown as mean values and standard deviation and analyzed with two-way ANOVA followed by Tukey’s test for the statistical determination of the differences.

When evaluating the post-freezing proliferation kinetics of the different cell types, it was observed that after 72 h of culture, there were no significant differences in the proliferation curve of the fibroblasts that had previously been frozen with the three freezing solutions based on PL, PLS or FBS, as can be seen in Figure 8A. In the case of the WJ-MSC culture, no significant differences were observed in the proliferation curve of the cells previously frozen with the solutions supplemented with PL and PLS at 72 h of culture. When comparing FBS with PLS, there were significant differences (*p* < 0.005) in favor of PLS (Figure 8B). Finally, more erratic behavior was observed in AdMSCs at 72 h, with a significant decrease in the proliferation of cells frozen with the PL-based freezing solution (*p* < 0.0001) (Figure 8C).

Based on the above, it can be stated that both the post-freezing cell viability and the proliferation of the cells evaluated in solutions supplemented with PLS were equivalent to or better than with solutions supplemented with FBS. PLS was a better substitute than PL for FBS as a standard cryopreservation and cell thawing additive in this study.

## 4. Discussion

In this study, concentrations of various biochemical components in platelet by-products were compared to concentrations of these in normal plasma levels of human blood; various growth factors and cytokines were analyzed in the PL, PLS, and FBS. In addition, the performance of cell culture media supplemented with these additives on the proliferation kinetics and the efficiency of freezing and thawing of different cell types were evaluated.

The comparison between PL and PLS showed significant differences in fibrinogen and Ca^2+^ concentrations between the two supplements, which can be attributed to the fact that clot formation was induced with 10% calcium gluconate in the PLS preparation (calcium: 0.301 mg/mL) to avoid the use of heparin of animal origin in the culture medium [22]. As expected, most PL and PLS biochemical parameters had concentrations similar to human reference blood values. Similar results to these were found in the study by Laner-Plamberger et al. [3], in which the biochemical composition of three different preparations of human PL derived from a PL batch consisting of 10 units of platelets of mixed ABO blood groups was evaluated and compared with FBS. Additionally, in that study, the high glucose and Na^+^ levels found were attributed to the addition of citrate phosphate dextrose (CPD) to the blood bags as an anticoagulant. In the present study, citrate phosphate dextrose adenine (CPDA-1) was added as an anticoagulant with high glucose concentration to the collection bags of platelets poor in leukocytes. The detection of higher Fe^3+^ levels in FBS than in PL and PLS in our study is consistent with the fact that bovine fetuses show elevated Fe^3+^ reserves [3]. 

Based on the results of this study, it can be stated that the preparation of PL and PLS from batches of 100 units of leukocyte-poor platelets enabled the achievement of homogeneous concentrations of the biochemical components analyzed, with minimal variability and reduced heterogeneity between batches. This was not observed when the concentrations of the components of the three commercial batches of FBS were compared, as the values of most of the biochemical components varied. In similar studies, Burnouf et al. [17], Hemeda et al. [1], and Cañas-Arboleda et al. [2] have highlighted that the use of platelets from allogeneic donors is an attractive option for the preparation of PL since it has several advantages over autologous preparations, such as low batch-to-batch variations due to donor pooling, higher volume, reproducibility of production, and affordable extraction [2].

Despite the numerous protocols available for obtaining platelet pools from different sources of collected platelets, there is no standardized consensus on the preferable type of platelet unit to produce PL batches. Platelet units can be collected by apheresis or by separation of blood components from autologous or allogeneic donors, either derived from buffy coats or leukocyte-poor platelets (LPP)collected from multiple donors of whole blood or platelet-rich plasma (PRP). In a study by Cañas-Arboleda et al. [2], three LPP units were used for each platelet pool batch prepared, and the platelet concentration was adjusted to 6 × 10^5^/µL to obtain more homogeneous PLP groups. However, in this case, the number of LPP units used to adjust and prepare each PL batch was much lower than the 100 units used in our study. Other authors, such as Horn et al. [28], Schallmoser et al. [24,25], Laner-Plamberger et al. [3,29], and Fernandez et al. [30] have used between 5 and 10 units of platelets concentrated from the buffy coats of different units of whole blood, using a different method for obtaining platelets than the one used in this study (LPP). 

Methods for preparation and supplementation of culture media are important because the supplement solutions used in practice can vary widely in quality, which can influence some of the evaluable aspects of cell culture (expansion kinetics, growth, morphology, and phenotype). On the other hand, commercial FBS and PL are expensive, and their prices can vary according to market dynamics, geopolitical factors, inflation, health legislation, demand, and other economic factors, all of which affect the value of cell production for research or therapeutic use and the quality of the final product. In this study, the Cellular Therapy and Biobank laboratory of the University of Antioquia and the Alma Máter Hospital in Antioquia developed their protocol to obtain a pool of platelets and their derivatives, PL and PLS, from batches of 100 units of PLP of blood types O, Rh + and −. The product obtained presented low variability in the concentrations of its components between different batches, high volume, reproducibility of production, optimization in the use of resources, and reduction in the cost of cell production. In addition, this method enables the use of a biological resource that would otherwise be considered a waste product and confirms the feasibility of using blood components not used in the clinic as a source of supplementation for culture media.

The concentrations of soluble factors representative of the platelet secretome present in PL and PLS were higher than those in commercial FBS. However, no significant differences were found in the concentrations of any of these factors between preparations derived from platelets. Although low fibrinogen levels and high calcium levels were detected in PLS, it was established that this difference did not have a statistically significant influence on the concentration of growth factors or immunoglobulin G, levels of which were found to be elevated in both PL and PLS. The factors identified here belong to the group of immunomodulatory molecules (IL-10, IL-6, TGF alpha, GM-CSF, and G-CSF) and the RANTES chemokine and growth factors (IGF-1, VEGF-A, PDFG -AA, AB, basic FGF, EGF, and TGF-β1) involved in cell stimulation, differentiation, maintenance, and growth. Concentrations of these factors were found to be significantly higher in the platelet-derived preparations than in the FBS.

Several factors make it difficult to compare the concentrations of growth factors and cytokines found in this study with those obtained in other studies. These are (I) the type of method used to obtain the platelet units, which includes platelet-rich plasma [19], LPP [2], apheresis [31], or derivation from buffy coats [28], since the obtained concentrations of platelets and growth factors and cytokines differ depending on the method; (II) the differences between the method used to measure the concentrations of these molecules (ELISA or Luminex) [3,16], as differences in techniques, rationale, and supplier can cause the values of the factors to differ [26]; (III) preparation methods are also different between studies and include repeated freeze/thaw cycles, direct platelet activation by calcium chloride, sonication or solvent/detergent treatment [21,24,31,32] and; (IV) the source material could be fresh, expired, irradiated or inactivated for pathogens [33], factors that could influence the quality of the final product and may not be known to users.

For these reasons, the lack of standardization in storage, selection, processing, and production storage of platelet derivatives can affect reproducibility and limit comparison between the results of clinical trials carried out by different groups, as highlighted by Guiotto et al. [34]. However, our results support the findings that platelet derivatives are a valuable product that contains high concentrations of various growth factors and cytokines and can serve as a supplement to cell growth media for WJ-MSCs, AdMSCs, and fibroblasts [2,3,16,26,35,36].

The advantages of using culture media supplemented with xeno-free derivatives, such as the PL and PLS used in this study, are the decreased risk of zoonoses and xenogenic immune reactions in the transplanted host, the reduction of cell production costs, the protection of animal welfare, the conservation of the livestock inventory by reducing the need to allocate bovine fetuses for the preparation of FBS and adherence to the regulations established by the competent authorities [31,32,37,38].

Various protocols have been described in the literature for the ex vivo culture and expansion of Wharton’s jelly mesenchymal stem cells (WJ-MSCs), adipose tissue (AdMSCs), and bone marrow MSCs that contain platelet derivatives as an alternative supplement to FBS. Platelet-derived growth factors have aroused great interest, given their ability to promote the ex vivo expansion of MSCs. Furthermore, according to Astori et al. [33], Becherucci et al. [37], and Bernardi et al. [31], MSCs expanded with PL have already been used clinically as cell therapies given that, as corroborated in this study, cell viability and proliferation are maintained when PL is used for this purpose. The restrictions on the production, distribution, and availability of FBS and its use in expanded preparations make the use of PL even more advantageous [1,38].

Human platelet components have become a feasible alternative to traditional cell culture supplements and have gained acceptance over the last few decades, which is due especially to their ability to promote cell proliferation and maintenance. In this study, we observed that the proliferative capacity of fibroblasts, WJ-MSCs, and AdMSCs cultured with platelet supplements was maintained or improved compared to media supplemented with traditional FBS. In the case of fibroblasts, Fernández et al. [30], used six bags (30 donors in total) of platelet concentrate that were close to their expiration date and carried out various protocols for the preparation of human PL, some involving combination with other commercial additives. Their objective was to evaluate the efficacy of cultured human fibroblasts with media supplemented with platelet derivatives in comparison with media supplemented with FBS. The results showed that the media supplemented with PL favored the expansion of fibroblasts and the maintenance of the normal phenotype and karyotype to the same extent as in the media supplemented with FBS and commercial PL. These findings are consistent with those of this study and suggest that platelet derivatives are a good alternative to fibroblast culture [30].

Unlike FBS, PL prepared from platelets contains thrombocyte-derived factors, fibrinogen, and coagulation factors that can lead to fibrin clot formation and cause culture media to gel. The most widely used strategy to avoid fibrin formation is perhaps the addition of heparin; however, this anticoagulant must be added in very low concentrations since high concentrations of heparin are cytotoxic and alter cell proliferation, as described in several reports [16,39]. As an alternative solution to this problem, in this study, it has been proposed to obtain serum from PL (PLS) using 10% calcium gluconate to induce coagulation and activate the coagulation factors present in the lysate, thereby obtaining a fibrinogen and fibrin-free supplement that does not require the use of heparin.

Finally, the implementation of MSC and fibroblast therapy for regenerative medicine requires the establishment of cryopreservation methods that allow cells to retain their viability, functionality, and phenotypic stability even after being subjected to freezing and thawing processes. Cryopreservation protocols are based on cryoprotective substances such as dimethyl sulfoxide (DMSO), glycerol, dextran, and FBS [40,41]. There are different protocols and studies on cryopreservation based both on xeno-free commercial solutions and on human platelet lysate in different cell types, mainly in MSCs from the umbilical cord, bone marrow, and adipose tissue. These have shown that DMSO combined with PL in different concentrations maintains cell viability and proliferation after post-thawing recovery and that PL can function as a substitute for FBS in cryopreservation solutions [42,43,44,45], a conclusion similar to that which can be inferred from the results of this study.

The findings of this study suggest that adding PL or PLS to DMSO-based cryopreservation and thawing solutions improves the viability and function of fibroblasts and WJ-MSCs after cryopreservation. In the case of AdMSCs, PLS- and FBS-based freezing solutions show similar results for cell viability percentage and proliferation; however, the effectiveness of PL-based freezing solutions is low, possibly due to the addition of heparin to the freeze–thaw solution, which affects to some extent the stability and cell proliferation of adipose tissue mesenchymal cells.

In a study by Taylor et al. [45], different formulations of heparin-free PL freezing media were compared to similar formulations containing FBS and commercially available serum-free freezing solutions. The objective was to evaluate PL without heparin as a freezing medium together with DMSO and as a post-thawing recovery medium for MSCs obtained from various tissues. Results showed high cell viability and normal proliferation after thawing of cells, which were similar to those of cells frozen with serum-free media and with freezing media containing FBS. These findings corroborate the feasibility of using heparin-free PL as a substitute for FBS in freezing solutions for MSCs [45].

Based on the previous results, it can be stated that freezing and thawing solutions of cells supplemented with PLS maintain cell viability and proliferation, and those are even higher when compared with solutions supplemented with FBS. This suggests that PLS is the best substitute for such solutions, not only in terms of efficacy but also in terms of availability and lower cost than FBS. As for LP, more studies are required to evaluate the role of heparin in the cryopreservation process for adipose tissue mesenchymal cells.

## 5. Conclusions

The use of platelet units deemed unsuitable for transfusion, donated by blood banks and pre-transfusion services, would result in savings related not only to the procurement of blood components and the supply of supplements but also to the infectious disease testing required before a platelet unit is considered suitable for transfusion. Consequently, this reduces costs associated with operational processes and cell production, both for therapeutic and research purposes. It also provides a secondary purpose for a biological product that would otherwise be discarded as common waste in blood banks. This approach is beneficial and contributes to a more efficient and sustainable management of biological resources.

Notably, the percentage of culture media supplementation with PL and PLS is 5%, representing half of the supplementation percentage compared to FBS, set at 10%. This difference could be attributed to the varied amount of growth factors and biochemical components present in the mentioned platelet derivatives, suggesting that the selection of the type of supplementation can significantly influence the conditions of cellular growth and development.

Obtaining a pool of platelet lysate from the units obtained from 100 donors decreased the variability between batches. The PLS made from this pool preserved the growth factors and other plasmatic components at levels that were similar to physiological ones and higher than those found in FBS, making PLS a suitable substitute for FBS as a supplement to media for the culture, expansion, and cryopreservation of cells. In addition, its incorporation in the media avoids the use of heparin, commonly used in media supplemented with PL. Therefore, the protocol developed from this study allows the “in-house” production of animal serum-free media, which results in decreased operating costs and greater safety for the use of cells in advanced therapies.

## Figures and Tables

**Table 1:** Freezing and thawing test of fibroblasts, WJ-MSC, and AdMSC.

Cryopreservation Solution	DMSO 10% + PL 90%		DMSO 10% + PLS 90%		DMSO 10% + FBS 90%	
	Replicas		Replicas		Replicas	
*Cellular type*	1	2	1	2	1	2
Fibroblasts	6.7 × 10^5^	6.7 × 10^5^	6.7 × 10^5^	6.7 × 10^5^	6.7 × 10^5^	6.7 × 10^5^
WJ-MSC	1.0 × 10^6^	1.0 × 10^6^	1.0 × 10^6^	1.0 × 10^6^	1.0 × 10^6^	1.0 × 10^6^
AdMSC	1.0 × 10^6^	1.0 × 10^6^	1.0 × 10^6^	1.0 × 10^6^	1.0 × 10^6^	1.0 × 10^6^
Cryopreservation time	7 days		7 days		7 days	
Thawing solution	70% DMEM/Ham-F12 + 30% PL		70% DMEM/Ham-F12 + 30% PLS		70% DMEM/Ham-F12 + 30% FBS	
Viability	Trypan blue		Trypan blue		Trypan blue	
Adhesion and proliferation	1.75 × 10^4^	1.75 × 10^4^	1.75 × 10^4^	1.75 × 10^4^	1.75 × 10^4^	1.75 × 10^4^
	Sowing for 72 h		Sowing for 72 h		Sowing for 72 h	

**Table 2:** Comparison of the biochemical parameters analyzed in the PL, PLS, and FBS preparations.

	Supplements			Reference Plasma Values
	PL	PLS	FBS	
Total proteins (gr/dL)	5.93 ± 0.2	5.73 ± 0.2	3.58 ± 0.2	6–7.8
Albumin (gr/dL)	3.89 ± 0.0	3.78 ± 0.1	2.61 ± 0.1	3.5–5.4
Transferrin (mg/dL)	206.28 ± 3.6 ***	206.28 ± 9.4 ***	0.1 ± 0.2	212–360
Ferritin (ng/mL)	89.62 ± 5.5 ***	86.97 ± 8.1 ***	1.04 ± 0.5	30–300
Fibrinogen (mg/dL)	141 ± 56.5 ***^, +++^	<50	<50	150–350
Immunoglobulins (mg/dL)	1038.16 ± 11.0 ***^, +^	1016.82 ± 31.4 ***^, +^	4.59 ± 9.1	640–1430
Total cholesterol (mg/dL)	152.14 ± 5.7 ***	141.81 ± 4.2 ***	32.58 ± 3.3	150–199
LDL (mg/dL)	103.48 ± 2.5 ***	96.25 ± 2.5 ***	20.4 ± 3.8	<130
HDL (mg/dL)	35.99 ± 1.2 ***	34.90 ± 0.9 ***	5.5 ± 0.3	>40
Triglycerides (mg/dL)	121.58 ± 16.1 ***	103.51 ± 9.1 ***	49.58 ± 34.8	<250
Glucose (mg/dL)	192.75 ± 23.1 ***	189.75 ± 21.0 ***	69.25 ± 51.2	70–110
Ca^2+^ (mg/dL)	7.55 ± 0.0 ^+++^	36.04 ± 2.2 *^, +++^	14.44 ± 0.3	9–10.5
Cl^−^ (mmol/L)	83.07 ± 2.4	80.55 ± 2.5*	103.50 ± 1.2	98–106
K^+^ (mmol/L)	4.26 ± 0.1	4.24 ± 0.2	10.71 ± 1.4	3.5–5
Na^+^ (mmol/L)	162.10 ± 4.8 *	159.13 ± 3.8 *	137.21 ± 1.4	136–145
Mg^2+^ (mg/dL)	1.87 ± 0.0	2.98 ± 0.1	3.32 ± 0.1	1.5–2.4
Fe^3+^ (μg/dL)	79.5 ± 2.6 ***	77.5 ± 5.3 ***	187.75 ± 20.7	60–160
Cortisol (μg/dL)	7.28 ± 0.7 ***	7.73 ± 1.6 ***	0.05 ± 0.1	3–20
Insulin (mUl/L)	2.61 ± 0.3 ***	2.57 ± 1.3 ***	0.06 ± 0.1	2.6–37.6
Triiodothyronine (ng/mL)	1.21 ± 0.1	1.08 ± 0.2	3.05 ± 3.3	0.7–1.95
Thyroxine (mg/dL)	7.28 ± 0.3 ***	7.18 ± 0.3 ***	15.6 ± 4.6	5–12
TSH (uIU/mL)	1.72 ± 0.2 ***	1.71 ± 0.3 ***	0.03 ± 0.0	0.5–5

Data are shown as mean ± standard deviation and analyzed with two-way ANOVA followed by Tukey’s test. FBS vs. PL o PLS: *** *p* < 0.0001; * *p* < 0.05; PL vs. PLS: ^+++^ *p* < 0.0001; ^+^ *p* < 0.05.

## Data Availability

The data presented in this study are available on request from the corresponding author.
